# Construction and Validation of a Ferroptosis-Related Prognostic Model for Endometrial Cancer

**DOI:** 10.3389/fgene.2021.729046

**Published:** 2021-09-28

**Authors:** Hao Wang, Yingchen Wu, Shengfu Chen, Minzhi Hou, Yanning Yang, Meiqing Xie

**Affiliations:** Department of Gynecology and Obstetrics, Sun Yat-sen Memorial Hospital, Sun Yat-sen University, Guangzhou, China

**Keywords:** ferroptosis, prognostic model, endometrial cancer, molecular characteristics, clinical characteristics

## Abstract

Endometrial cancer (EC) is one of the most common female reproductive system tumors, with close to 200,000 new cases each year. It accounts for approximately 7% of the total number of female cancers, but until now the cause of EC has remained unclear. Ferroptosis is regulated cell death that distinguishes apoptosis and caused by oxidative damage. The process has unique biological effects on metabolism and redox biology. In this study, we analyzed the relationship between EC and ferroptosis. According to the different expression levels of related genes, we first divided 544 EC samples into four clusters and found that most of the infiltrating immune cells were significantly different among the four groups. A differential gene expression analysis between Fe.cluster groups was performed, and the samples were again divided into three Fe.gene.cluster groups. The molecular characteristics and clinical characteristics of the groups were significantly different. Finally, 13 characteristic genes were selected as ferroptosis gene signatures, and the Fe.score was obtained by calculation. The Fe.score is closely related to the clinical and molecular characteristics of EC, and a low Fe.score has a significant survival advantage. The GDSC predicts that the IC50 of multiple chemotherapeutic drugs is also significantly different between the two groups. In conclusion, our research has explored the relationship between EC and ferroptosis in detail, provides comprehensive insights for ferroptosis-mediated EC mechanism research, and emphasizes the clinical application potential of Fe.score-based immunotherapy strategies.

## Graphical Abstract

Analysis workflow chart.

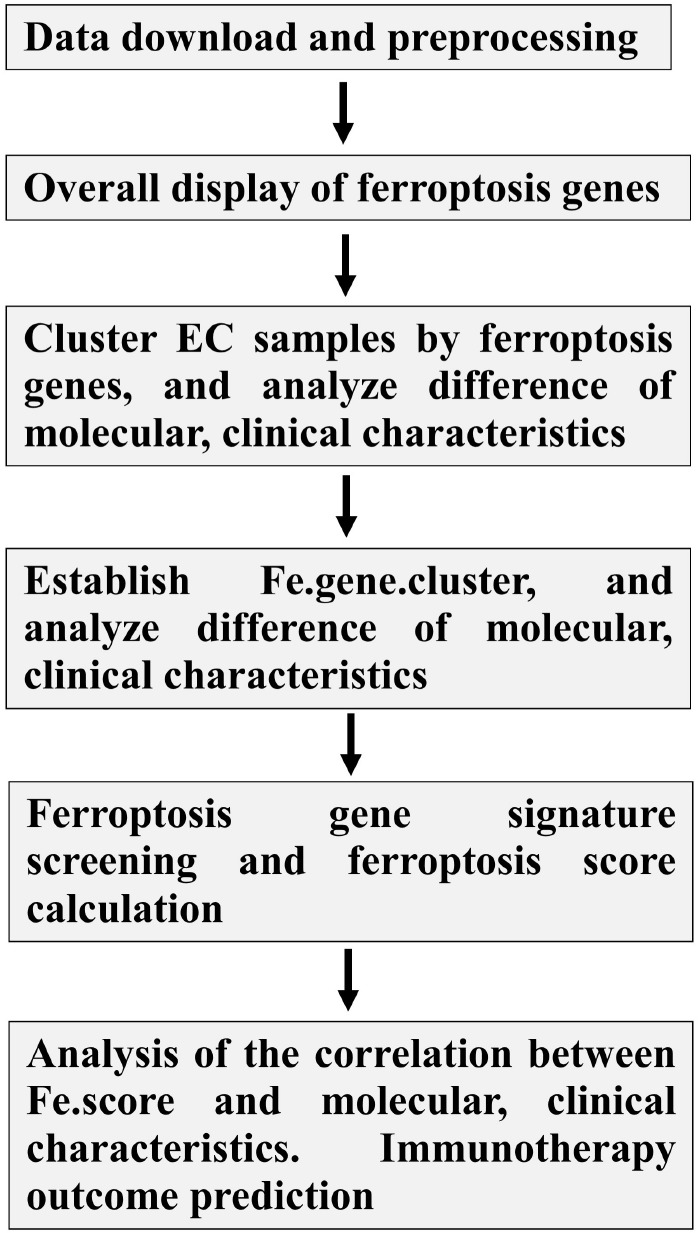

## Introduction

Endometrial cancer (EC) is one of the most common types of gynecological malignancies, and it affects the health of many women around the world. As the eighth leading cause of cancer-related deaths in women, the morbidity and mortality of EC are increasing rapidly (Makker and Goel, 2016; Henley et al., 2020). For patients with metastasis or recurrence, the prognosis is unfavorable. These patients have a significantly higher risk of death and always have a low quality of life (Lu and Broaddus, 2020). Obesity is one of the most important risk factors for this disease, and other recognized risk factors for EC include long-term exposure to endogenous or exogenous estrogen, age at menopause, age at menarche, history of infertility, polycystic ovary syndrome, diabetes, and previous pelvic radiotherapy. In the past few years, the surgical treatment of EC has improved significantly. Now, in addition to minimally invasive removal of the uterus, ovarian tube, and fallopian tube, sentinel lymph node mapping is performed. Data from The Cancer Genome Atlas (TCGA) project have advanced our understanding of the biological heterogeneity of EC (Liu et al., 2014; Cherniack et al., 2017). Recently, a study integrated the sequencing of eight omics approaches among the four genomic subtypes of EC, providing valuable resources for researchers and clinicians to identify new molecules with potential diagnostic and therapeutic significance in the development of EC (Dou et al., 2020). Although we have gained a great understanding of the molecular characteristics of EC, at present, it is still difficult to predict the prognosis of EC patients and seek convenient and effective biomarkers.

Ferroptosis is a newly discovered iron-dependent cell death that is different from other forms of cell death, including apoptosis and necrosis. Ferroptosis involves three main metabolites, thiol, lipid, and iron, leading to iron-dependent lipid peroxidation and ultimately cell death (Yan et al., 2021). Ferroptotic cell death is accompanied by a series of changes in cell morphology, metabolism, and protein expression, which can be distinguished from other forms of cell death. At the cellular and subcellular levels, cells undergoing ferroptotic action have a characteristic round shape before death, similar to necrotic cells, but without swelling of the cytoplasm and organelles or plasma membrane rupture (Yagoda et al., 2007). The nucleus of ferroptotic cells maintains its structural integrity without condensation, marginalization of chromatin, plasma membrane blistering, or apoptotic body formation (Dixon et al., 2012), which is a characteristic feature of apoptosis. The only unique morphological feature is mitochondria, which appear to be smaller than normal and have an increased membrane density (Dixon et al., 2012).

Ferroptosis is involved in the occurrence and development of many diseases, including neurodegenerative diseases, such as Alzheimer’s disease, Parkinson’s disease (PD) (Belaidi and Bush, 2016), and ischemia/reperfusion (Scindia et al., 2015; Bulluck et al., 2016), and most importantly, it is closely related to various types of tumors. Compared with normal cells, cancer cells have a higher iron content (Spangler et al., 2016). Current studies have found abnormalities in iron homeostasis in a variety of cancers, including breast cancer, ovarian cancer, and lung cancer (Spangler et al., 2016). Ferroptosis is involved in many important pathways in tumors. P53 is one of the most important suppressor genes in the human body and is biallelically mutated or deleted in approximately 50% of all human cancers (Joerger and Fersht, 2016). P53-mediated transcriptional suppression of SLC7A11 promotes ferroptosis in cancer cells (Joerger and Fersht, 2016). P53 3KR (K117R, K161R, and K162R) acetylation-deficient mutants cannot induce apoptosis but completely retain the ability to induce ferroptosis in lung cancer cell lines (Jiang et al., 2015). In addition, oncogenes of the *RAS* family (*HRAS*, *NRAS*, and *KRAS*) are the most commonly mutated in all human cancers (Ryan and Corcoran, 2018). The ferroptosis-inducer erastin has shown selective lethality against engineered *RAS*-mutant tumor cells (Dolma et al., 2003). *KRAS*-mutant lung adenocarcinoma cells are susceptible to SLC7A11 inhibitor-induced ferroptosis (Hu et al., 2020); in addition, NSCLC-derived cells with upstream mutations in *EGFR* are sensitive to ferroptosis (Poursaitidis et al., 2017). Ferroptosis is also closely related to the expression of nuclear factor, erythroid 2 like 2 (Sun et al., 2016), hypoxia-inducible factor (HIF) (Cho et al., 2013; Ivan and Kaelin, 2017), and important processes such as epithelial–mesenchymal transition (EMT) in tumors (Viswanathan et al., 2017)

Ferroptosis is currently involved in many antitumor therapies, including immune and radiation therapy. Cytotoxic T cell-driven immunity can induce ferroptosis in cancer cells. Anti-PD-L1 antibodies can promote hypertrophy in tumor cells, and the hypertrophy inhibitor liproxstatin 1 reduces the anticancer activity of these drugs (Wang et al., 2019). In addition, anti-PD-L1 antibodies and ferritin activators (such as erastin and RSL3) synergistically induce tumor growth inhibition (Wang et al., 2019). The antitumor effect of radiation is attributed to the particles released by the irradiated cells, which have been shown to induce immunogenic death mainly through ferroptosis (Wan et al., 2020). Treatment with erastin in HeLa and NCI-H1975 adenocarcinoma cell lines aggravates radiation-induced cell death (Shibata et al., 2019). Ferroptosis inducers combined with temozolomide and haloperidol can enhance the chemotherapeutic effects of these drugs in tumor treatment (Chen et al., 2015; Bai et al., 2017). In addition, some new ideas and technologies, such as nanotechnology, can be combined with ferroptosis to treat tumors. A new kind of nanoparticle has been used to treat brain tumors *in situ* by delivering Fe^2+^ and Fe^3+^ (Shen et al., 2018). Upconversion nanoparticles, which induce ferroptosis, were invented and tested in 4T1 xenograft mice (Bao et al., 2019).

However, at present, there is very little research on the role of ferroptosis in EC. In this study, we analyzed the different molecules related to ferroptosis in EC, reveal the correlations between ferroptosis and EC, and explore whether there are potential diagnostic and therapeutic target molecules.

## Materials and Methods

### Data Download and Preprocessing

The somatic mutation, transcriptome, CNV, and sample phenotype data of EC were downloaded from the TCGA xena database. Among them, there were 530 samples with somatic mutation data; there were two sets of CNV data, one of which consisted of the CNV results compiled by xena, showing CNV at the gene level, and the other was the sample DNA copy file downloaded from http://portal.gdc.cancer.gov/, which is convenient for grouping and performing later gistic2 analysis. One set of RNA-Seq data, labeled TCGA-EC, consists of a total of 583 samples. After removing four samples without survival information, the expression data of 579 samples were finally obtained, including 544 tumor tissues (clinical information shown in Table 1) and 35 normal tissues. The downloaded expression profile data format was log2(FPKM + 1). The expression value was restored to FPKM by the formula FPKM = 2^original^ ^expression value^ −1 and then converted to TPM by the following formula: and finally, log2(TPM + 1) conversion was performed. This data was used for subsequent analysis.

**TABLE 1:** TCGA-EC samples’ clinical information.

	**TCGA-UCEC**
Number of sample	544
Age (median, range)	64 (31–90)
Stage (%)	
I	338 (62.1%)
II	51 (9.4%)
III	126 (23.2%)
IV	29 (5.3%)
Grade (%)	
G1	98 (18.0%)
G2	119 (21.9%)
G3	316 (58.1%)
High grade	11 (2.0%)
Survival status	
OS (sample)	544
OS (median)	909.5
Censored (%)	452 (83.1%)
Diabetes (%)	
YES	100 (18.4%)
NO	267 (49.1%)
Not reported	177 (32.5%)
Hypertension (%)	
YES	232 (42.6%)
NO	161 (29.6%)
Not reported	151 (27.8%)
Pregnancies (%)	
0	65 (11.9%)
1	51 (9.4%)
2	116 (21.3%)
3	67 (12.3%)
4+	74 (13.6%)
Not reported	171 (31.4%)
Radiation therapy (%)	
YES	224 (41.2%)
NO	286 (52.6%)
Not reported	34 (6.2%)
BMI (median, range)	32.21 (17.36–213.5)


$${\text{TPM}}_{i}=\frac{{\text{FPKM}}_{i}\times 1000000}{\sum_{i=1}^{n}{\text{FPKM}}_{i}}$$

The gene sets of 23 infiltrating cells were obtained from a reference (Zhang B. et al., 2020). The gene sets of angiogenesis, CD8 T effector, EMT1, EMT2, EMT3, and panfibroblast TGFb were downloaded from another reference (Mariathasan et al., 2018). From an additional reference (Liang et al., 2020), 60 ferroptosis genes were obtained (see Supplementary Table 1). The c2.cp.kegg.v7.1.symbols with a total of 186 gene sets were downloaded from MsigDB.

### Overall Display of Ferroptosis Genes

Using TCGA-EC RNA-Seq data, the expression values of 60 ferroptosis genes were extracted, and the R package *ggpubr* was used to draw box plots to show the differences in the expression of these genes between tumor tissues and normal tissues. The CNV results of ferroptosis genes were extracted, the frequency of amplification and deletion was counted, and a dot plot was drawn. The R package *maftools* was used to import the maf files of somatic mutations in 530 EC samples and draw the somatic mutation spectrum. The R package *RCircos* was used to draw a circos map of 60 ferroptosis genes, showing the positions of the genes on the reference genome. The R package *pca3d* was used to perform principal component analysis (PCA) on the expression matrix of 60 ferroptosis genes and draw a three-dimensional PCA map.

### Ferroptosis Cluster

Using the expression matrix of ferroptosis genes in 544 EC samples as input files, the R package *ConsensusClusterPlus* was used to perform unsupervised clustering with the following parameters: *m* (maximum number of categories) = 6, reps (repeated sampling) = 1,000, pItem (proportion of items selected each time) = 0.8, pFeature (the proportion of features selected each time) = 1, the clusterAlg (clustering algorithm) = “pam”, distance (calculation distance) = “spearman.” The output results were synthesized, the *K* values were filtered, and the classification of each sample was obtained.

### Gene Set Variation Analysis Enrichment Function Analysis

For Fe.cluster, grouped two by two, the expression matrix of all genes of the two samples was extracted, the c2.cp.kegg.v7.1.symbols gene set was combined as the input file, gene set variation analysis [GSVA; a GSE method that estimates the variation of pathway activity over a sample population in an unsupervised manner (Hänzelmann et al., 2013)] was performed, and the enrichment score of each sample for each gene set was obtained. Then, the R package *limma* was used to analyze the differences in the gene sets, and the threshold was a BH-corrected *p*-value < 0.05. The top 20 differential gene sets were extracted, the R package ComplexHeatmap was used to draw the differential gene set heat map, and group labels were added.

### Assessment of 23 Types of Infiltrating Immune Cell Ratios and Differences by Single-Sample GSEA

The gene expression matrix of 544 tumor samples and the gene set of 23 infiltrating cells were used as the input files of the R package GSVA for single-sample GSEA (ssGSEA). Using the enrichment score as the content of each cell, a box plot was drawn using the R package ggpubr, and the Kruskal–Wallis rank sum test was performed to show the differences in the content of 23 infiltrating cells between the Fe.cluster groups. Differential cells were selected, the R package *survival* was used to perform single-factor Cox risk regression analysis to obtain the hazard ratio (HR) and *p*-values of differential cells, and then the R package *forestplot* was used to draw forest plots to visually display the prognostic effects of differential cells.

The display of angiogenesis, CD8 T effectors, EMT1, EMT2, EMT3, and panfibroblast TGFb enrichment scores between different Fe.cluster groups was also performed for ssGSEA following the method described above; the enrichment score of each sample was obtained for these six biological functions, the R package *ggpubr* was used to draw box plots, and the Kruskal–Wallis rank sum test was performed.

### Differential Gene Screening and Enrichment Analysis

The Fe.clusters were grouped in pairs, the expression matrix was extracted, and the R package *limma* was used for differential gene analysis, with a differential gene screening threshold of abs[log2(fold change)] > log2(1.25) and BH correction *p*-value < 0.05. Multiple sets of differential genes were obtained, and the intersection was taken as the final differential gene. The R package *clusterProfiler* was used for Gene Ontology (GO) and Kyoto Encyclopedia of Genes and Genomes (KEGG) enrichment analysis, and the screening threshold was BH correction *p*-value < 0.05 and *Q*-value < 0.05. The top 20 enrichment items of biological processes (BP), cell components (CC), molecular function (MF), and KEGG were selected, and a bubble chart was drawn to display the results.

### Ferroptosis.Gene.cluster

The differential gene expression matrix of 544 tumor samples was extracted as the input file, and the R package *ConsensusClusterPlus* was used to perform unsupervised clustering. The parameters were set as follows: *m* = 6, reps = 1,000, pItem = 0.8, pFeature = 1, clusterAlg = “pam,” and distance = “spearman.” The output results were synthesized, the *K* values were filtered, and the classification of each sample was obtained. Sample clinical data were integrated, survival analysis was performed on Fe.gene.cluster, and Kaplan–Meier curves were drawn. The expression matrix of 60 ferroptosis genes was extracted, and the R package *ggpubr* was used to draw a box plot to show the expression in different Fe.gene.clusters and perform the Kruskal–Wallis rank sum test.

### Ferroptosis Gene Signature Screening

For differentially expressed genes, the R package *survival* was used to perform single-factor Cox risk regression analysis, and genes were screened according to a *p*-value < 0.05. For the retained genes, the R package *randomForestSRC* was used to construct a random forest model, and then important feature variables were screened as ferroptosis gene signatures.

### Ferroptosis Score Calculation

The expression matrix of ferroptosis gene signatures was selected, PCA was performed, the two principal components were selected as PC1 and PC2, and the ferroptosis score (Fe.score) was calculated according to the following formula:


$$\text{Fe}.\text{score}=\sum \left({\mathrm{PC1}}_{\text{i}}+{\mathrm{PC2}}_{\text{i}}\right)$$

Sample clinical data were integrated and divided into high Fe.score and low Fe.score according to the median Fe.score. The R package *survival* was used to analyze the survival of the Fe.score, and the R package *survivalROC* was used to draw a 5-year ROC curve to evaluate the Fe.score survival model.

### Analysis of the Correlation Between Fe.score and Pathway Function

The ferroptosis gene and the top 10 differential gene sets of the three groups of GSVA were integrated to perform ssGSEA, and then the enrichment score was combined with the Fe.score to calculate the Pearson correlation coefficient matrix and calculate the *p*-value. The R package corrplot was used to draw the correlation diagram, and a *p*-value < 0.01 was considered to be extremely significant. Dots of the corresponding colors were drawn in the figure according to the correlation coefficient.

### Immunotherapy Outcome Prediction

The R package *pRRophetic* was used to perform GDSC drug IC50 prediction, and a box plot was drawn to show the IC50 difference between Fe.score groups. The bladder cancer data set (IMvigor210) (Geeleher et al., 2014) was used to calculate the Fe.score, and the differences in immunotherapy results between groups were analyzed. The model in this paper was compared with another model (Deng et al., 2020), and the R package *survivalROC* was used to draw a 5-year ROC curve to evaluate the Fe.score survival model.

### Statistics and Drawing Methods

The comparison of the two groups in the box plot in this paper uses the nonparametric Wilcox rank sum test; the comparison of multiple groups uses the Kruskal–Wallis rank sum test. The R package *ggalluvial* was used to draw the Fe.cluster, Fe.gene.cluster, and Fe.score grouping of 544 samples and survival status mulberry charts. The R package maftools was used to draw the somatic mutation map and CNV peak map, and the Gistic2 analysis result file of the sample was required when drawing CNV. However, there was no complete Gistic2 result in the TCGA database, so DNA copy was used to perform Gistic2 analysis first. For analysis methods and parameter settings, refer to http://docs.gdc.cancer.gov/Data/Bioinformatics_Pipelines/CNV_Pipeline/. The R package *ComplexHeatmap* was used to draw heat maps. The Spearman correlation coefficient between ferroptosis genes was calculated, and screening was performed according to a *p*-value < 0.001 and an absolute value of the correlation coefficient greater than 0.2. Using consensus clustering to cluster genes, single-factor Cox risk regression was used to determine the prognostic effects of genes, and the results were sorted into tables and imported into Cytoscape (3.7.2) to draw gene interaction network diagrams.

## Results

### Using TCGA Data to Comprehensively Display Ferroptosis Genes

**FIGURE 1:**
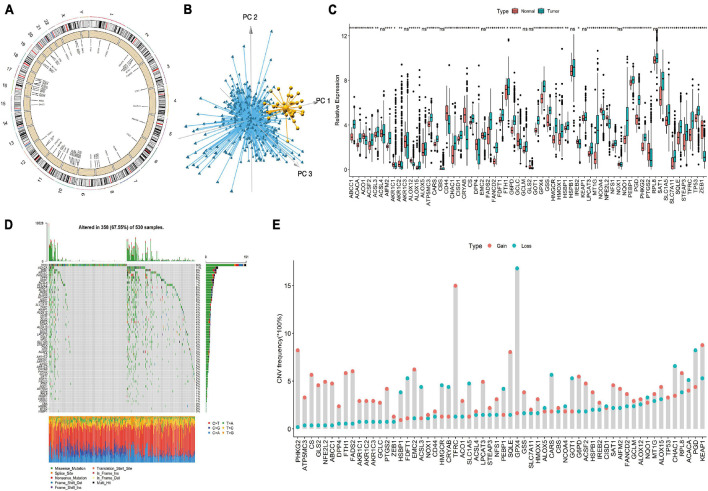
Display of ferroptosis genes in endometrial cancer (EC) samples. **(A)** The genomic positions of 60 ferroptosis genes. **(B)** Principal component analysis of 60 ferroptosis genes in tumor and normal samples. Blue, EC samples; yellow, normal samples. **(C)** The expression differences of 60 ferroptosis genes between tumor tissues and normal tissues. **(D)** Somatic mutation spectrum of ferroptosis genes in EC samples. **(E)** CNV mutation frequency of 60 ferroptosis genes in EC samples. Ns, *p* > 0.05; *p < 0.05; **p < 0.01; ***p < 0.001; ****p < 0.0001.

We first screened 60 ferroptosis genes, and the positions of 60 ferroptosis genes in the reference genome hg38 are shown in Figure 1A. Then, we analyzed the expression of the genes and found that, using the expression values of 60 ferroptosis genes for PCA, normal tissues and EC tissues could be clearly distinguished (Figure 1B). Figure 1C shows the detailed gene expression differences between EC tissues and normal tissues. Except for the differences in the expression of the ACSL4, AKR1C3, ALOX5, CBS, EMC2, GCLM, GLS2, HSPB1, KEAP1, NOX1, and RPL8 genes, which were not significantly different between normal tissues and cancer tissues, the expression of the other genes was significantly different. We further studied the mutations of ferroptosis genes and found many missense mutations in the TP53 gene in the EC samples (Figure 1D). For the CNV mutation frequency of the ferroptosis gene, we found that GPX4, PGD, and CHAC1 had a higher frequency (16.8, 8.2, and 6.6%) of fragment deletions, and TFRC, KEAP1, PHKG2, and SQLE had a higher frequency (15.0, 8.8, 8.2, and 8.0%) of fragment amplifications (Figure 1E).

### Endometrial Cancer Samples Were Clustered Into Four Groups by Ferroptosis Gene Expression and the Differences in Immune Infiltration in Each Group Were Explored

**FIGURE 2:**
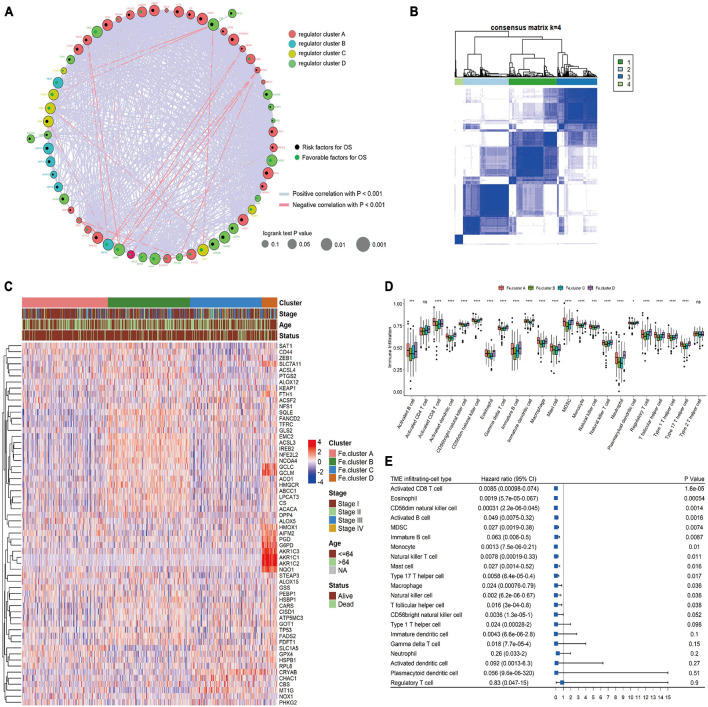
Cluster analysis of ferroptosis genes in endometrial cancer (EC) samples. **(A)** Ferroptosis gene interaction network diagram. Nodes of different colors represent genes of different categories; the node size corresponds to the log-rank test *p*-value of Cox risk regression analysis. The more significant the prognostic effect is, the larger the node. The green dots indicate good prognostic factors, and the black dots indicate prognostic risk factors. **(B)** Consensus clustering of the expression values of 60 ferroptosis genes in 544 EC samples. **(C)** Expression heat map of ferroptosis genes in Fe.clusters. **(D)** Box plot showing the difference in the proportion of 23 infiltrating cells in different Fe.clusters. **(E)** The prognostic forest plot of differentially infiltrated cells. Each row represents a type of infiltrated cell. The third column graphically displays the distribution of hazard ratios (HRs) in the 95% confidence interval. The value of the abscissa represents the HR. Ns, *p* > 0.05; *p < 0.05; **p < 0.01, ***p < 0.001; ****p < 0.0001.

We first performed cluster analysis of ferroptosis genes and divided them into four clusters: regulator cluster A, regulator cluster B, regulator cluster C, and regulator cluster D (see Supplementary Table 1 for the detailed results of the ferroptosis clusters). GPX4, SAT1, and TP53 in the ferroptosis gene regulatory network were significant prognostic protective factors. The CBS, CHAC1, and CISD1 genes were significant prognostic risk factors (see Supplementary Table 1 for details). Then, the correlation coefficient between ferroptosis genes was calculated, and statistical tests were performed. When the *p*-value < 0.001 and the absolute value of the correlation coefficient was greater than 0.2, the genes were considered to have an interaction relationship. As shown in Figure 2A, the larger the correlation coefficient, the thicker the connected line.

**FIGURE 3:**
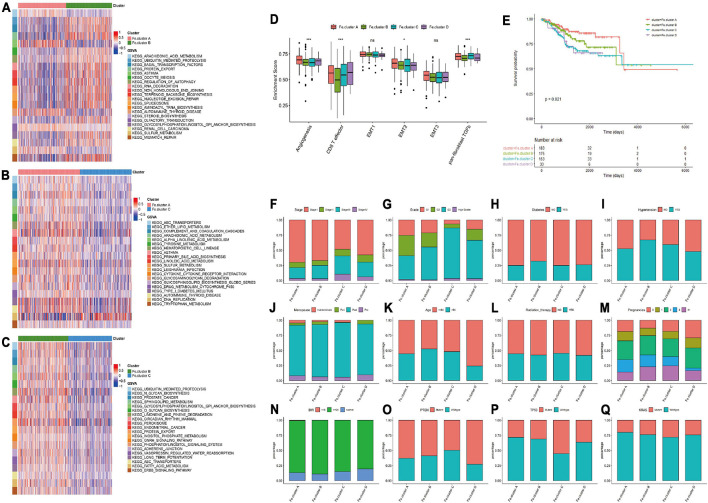
Molecular and clinical characteristics of clusters. **(A)** The top 20 differential enrichment pathways of Fe.cluster A and Fe.cluster B. **(B)** The top 20 differential enrichment pathways of Fe.cluster A and Fe.cluster C. **(C)** The top 20 differential enrichment pathways of Fe.cluster B and Fe.cluster C. **(D)** The differences in the enrichment scores of the main tumor-related biological processes in different Fe.clusters. **(E)** Kaplan–Meier survival curve of Fe.clusters. **(F–Q)** The distribution of stage, grade, diabetes, hypertension, menopausal status, age, radiotherapy status, fertility status, BMI, PTEN, P53, and KRAS mutation status between the Fe.cluster groups. *p < 0.05 and ***p < 0.001.

Then, we performed a cluster analysis of 544 EC samples based on the expression values of 60 ferroptosis genes (see Supplementary Table 2 for the classification results) and finally divided the samples into four clusters. In Figure 2B, 1, 2, 3, and 4 correspond to Fe.cluster A, Fe.cluster B, Fe.cluster C, and Fe.cluster D, and the number of samples in each cluster are 183, 175, 153, and 33, respectively. As shown in Figure 2C, most of the ferroptosis genes were highly expressed in Fe.cluster B. The expression pattern of the ferroptosis gene in Fe.cluster D was quite different from that in the other three groups, and the expression of part of the ferroptosis gene (PGD, G6PD, AKR1C1, AKR1C2, AKR1C3, NQO1) was particularly high in Fe.cluster D.

Then, we explored how the Fe.clusters reflect the immune status of EC. As shown in Figure 2D, 21 kinds of immune cells demonstrated significant differences in different Fe.clusters except for activated CD4 T cells and type 2 T helper cells. We also observed that activated CD8 T cells, eosinophils, CD56dim natural killer cells, and activated B cells were good prognostic factors for EC (Figure 2E).

### Fe.clusters Have Different Molecular and Clinical Characteristics

We next analyzed the specific molecular characteristics of Fe.clusters. The GSVA functional enrichment analysis results of Fe.cluster A, Fe.cluster B, and Fe.cluster C are shown in Figures 3A–C. It can be seen that most of the pathway enrichment scores of Fe.cluster B are higher than Fe.cluster A, and Fe.cluster A is higher than Fe.cluster C. We also focused on analyzing several key BP, and we found that the enrichment scores of angiogenesis, CD8 T effector, EMT2, and panfibroblast TGFb (Pan-F-TBRS) were significantly different in Fe.clusters (Figure 3D).

Then, we explored the relationship between clusters and clinical indicators. Figure 3E survival curve shows the survival difference between Fe.cluster groups: the log-rank test *p*-value is 0.021, the difference is significant, and Fe.cluster A has a higher survival time than the other three groups. In addition, as shown in Figures 3F–Q, for tumor stage, the proportion of Fe.cluster A and Fe.cluster B stage I patients is higher than the other two groups; for tumor grade, the proportions of G1 and of G2 patients in Fe.cluster A and Fe.cluster B are higher than in the other two groups. The proportion of patients with diabetes, hypertension, and more pregnancies in Fe.cluster A is lower than in the other three groups; menopausal status, age, radiotherapy, and BMI were not significantly different between the four groups. In Fe.cluster C, patients with PTEN mutation, TP53 wild-type, KRAS wild-type, and APC wild-type accounted for lower proportions than in the other three groups.

### Analysis of the Differential Genes Among Fe.clusters, Enrichment Analysis and Clustering to Obtain Different Fe.gene.clusters, and Analysis of the Characteristics

**FIGURE 4:**
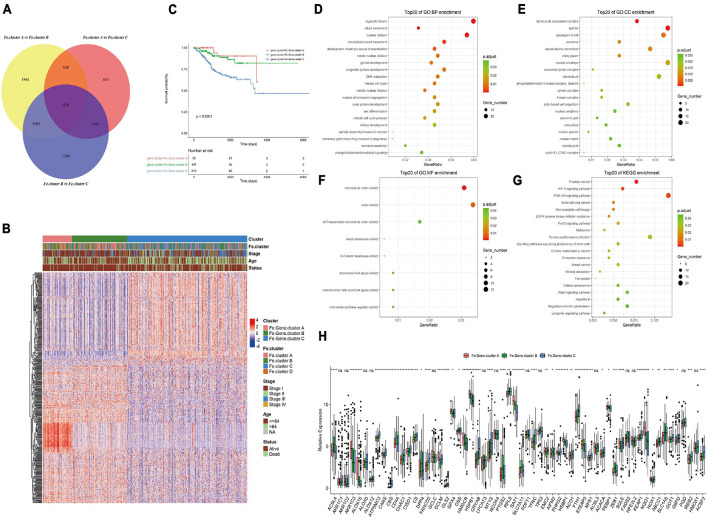
Molecular expression and prognosis of Fe.gene.clusters. **(A)** Total of 438 overlapping differential genes obtained from Fe.clusters. **(B)** Expression heat map of 438 overlapping differentially expressed genes among Fe.clusters. **(C)** Kaplan–Meier survival curve of Fe.gene.clusters. **(D)** The Gene Ontology (GO) enrichment results of biological processes of overlapping differentially expressed genes in ferroptosis clusters. The color indicates a significant degree of enrichment, the size of the bubble indicates the number of differential genes enriched in the pathway, and the abscissa indicates the proportion of genes. **(E)** The GO enrichment results of cell components. **(F)** The GO enrichment results of molecular function. **(G)** The KEGG enrichment pathways. The color indicates the significant degree of enrichment, the size of the bubble indicates the number of differential genes enriched in the pathway, and the abscissa indicates the proportion of genes. **(H)** The box plot shows the expression differences of 60 ferroptosis genes among the Fe.gene.cluster groups. Ns, *p* > 0.05, *p < 0.05; **p < 0.01; ***p < 0.001; ****p < 0.0001. **(C)** Kaplan–Meier survival curve of Fe.gene.cluster.

Considering that the ferroptosis gene expression pattern and clinical features of Fe.cluster D are quite different from those of the other three groups and the number of samples in this group is relatively small, a difference analysis only on the samples of Fe.cluster A, Fe.cluster B, and Fe.cluster C was performed, and 438 overlapping differential genes were obtained (see Figure 4A and Supplementary Table 3). Then, using the expression matrix of differential genes to perform consensus clustering, three groups were obtained, namely, Fe.gene.cluster A, Fe.gene.cluster B, and Fe.gene.cluster C. The sample numbers were 78, 147, and 319, respectively (see Supplementary Table 2 for the results of the Fe.gene.cluster). The expression heat map of 438 differentially expressed genes is shown in Figure 4A, and we can also see the relationship between Fe.gene.clusters and clinical indicators (stage, age, and status) from Figure 4B. Furthermore, we found that there was a very significant difference in survival between the Fe.gene.cluster groups, and Fe.gene.cluster A and Fe.gene.cluster B had obvious survival advantages (Figure 4C).

We continued to analyze 438 overlapping genes, and the GO and KEGG enrichment results are shown in Figures 4C–F. Differentially expressed genes were mainly involved in BPs such as organelle fission and cilium movement (Figure 4D), CCs such as microtubule-associated complexes and spindles (Figure 4E), MFs such as microtubule motor activity and motor activity (Figure 4F), and KEGG enrichment pathways such as prostate cancer and the HIF-1 signaling pathway (see Figure 4G and Supplementary Table 4 for the detailed GO and KEGG enrichment pathways).

Finally, we analyzed the differences in the expression of 60 ferroptosis genes between different Fe.gene.clusters. The expression differences of 14 ferroptosis genes in the Fe.gene.cluster group were not significant, and the remaining genes were significantly different (see Figure 4H).

### Screening Ferroptosis Gene Signatures and Calculating the Ferroptosis Score

Univariate Cox hazard analysis was performed on 438 differentially expressed genes, and according to *p* < 0.05, 204 statistically significant prognosis-related genes were screened. These 204 genes were used to build a random forest model and screen out 13 characteristic genes as ferroptosis gene signatures. PCA and calculation of the Fe.score were performed (see Supplementary Table 2 for details).

**FIGURE 5:**
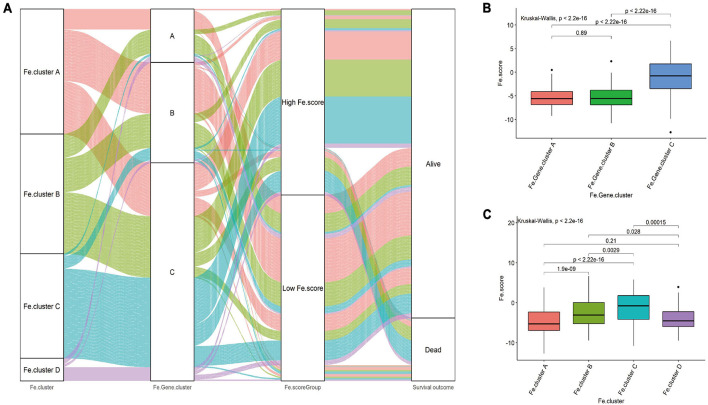
Correlation between Fe.scores, Fe.clusters, and Fe.gene.clusters. **(A)** The Sankey diagram shows the grouping of 544 EC samples. **(B)** The difference in Fe.score between Fe.gene.cluster groups. **(C)** Fe.score difference between Fe.cluster groups.

Then, we explored the relationship between Fe.score, Fe.gene.clusters, and Fe.clusters. Figure 5A shows that most samples of Fe.cluster C and Fe.cluster D correspond to Fe.gene.cluster C, and most samples of Fe.gene.cluster C belong to the high-Fe.score group, which has a survival disadvantage. Most samples of Fe.gene.cluster A and Fe.gene.cluster B belong to the low-Fe.score group, which has a survival advantage.

For a more detailed analysis, among the Fe.gene.cluster groups, the Fe.score of Fe.gene.cluster A and Fe.gene.cluster B was significantly lower than that of Fe.gene.cluster C (Figure 5B). Among the Fe.cluster groups, the Fe.score of Fe.cluster A and Fe.cluster B is extremely significantly lower than that of Fe.cluster C, and the difference between Fe.cluster A and Fe.cluster D is not significant (Figure 5C).

### Significant Differences in Pathway Functions and Molecular Characteristics Between the High- and Low-Fe.score Groups

**FIGURE 6:**
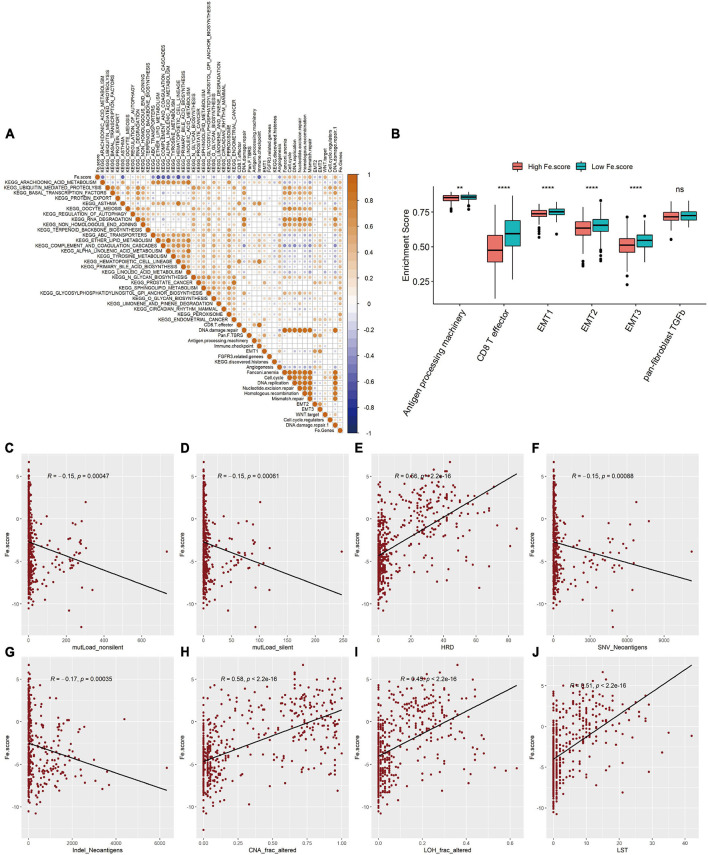
Correlation between ferroptosis score and molecular characteristics. **(A)** Correlation diagram between Fe.score and differential enrichment pathways. The color depth represents the degree of correlation: yellow represents a positive correlation, and blue represents a negative correlation, and white represents irrelevance. The size of the dot indicates the degree of correlation; the greater the absolute value of the correlation, the larger the point. *p*-Value < 0.01. **(B)** Differences in the enrichment scores of related biological processes among the Fe.score groups. **(C)** Correlation between Fe.score and mutLoad_nonsilent. **(D)** Correlation between Fe.score and mutLoad_silent. **(E)** Correlation between Fe.score and HDR. **(F)** Correlation between Fe.score and SNV_Neoantigens. **(G)** Correlation between Fe.score and Indel_Neoantigens. **(H)** Correlation between Fe.score and CNA_frac_altered. **(I)** Correlation between Fe.score and LOH_frac_altered. **(J)** Correlation between Fe.score and LST. MutLoad_nonsilent and mutLoad_silent indicate TMB, SNV_Neoantigens and Indel_Neoantigens indicate neoantigen load, and CNA_frac_altered, LOH_frac_altered, and LST indicate the level of chromosomal instability. **p < 0.01 and ****p < 0.0001.

We first explored the correlation between the Fe.score and cell function enrichment pathways. Figure 6A shows that the Fe.score is positively correlated with mismatch repair and DNA damage repair 1 and is negatively correlated with CD8 T effectors and immune checkpoints. Further statistical analysis shows that the enrichment scores of antigen processing machinery, CD8 T effector, EMT1, EMT2, and EMT3 all have extremely significant differences in the different Fe.score groups, and the Low Fe.score is higher than the High Fe.score (Figure 6B). In addition, for some new molecular indicators, Fe.score is significantly positively correlated with HRD, CNA_frac_altered, LOH_frac_altered, and LST, and the correlation coefficient is approximately 0.5; Fe.score is significantly negatively correlated with mutLoad_nonsilent, mutLoad_silent, SNV_Neoantigens, and Indel_Neoantigens, and the correlation coefficient is about −0.15 (Figures 6C–J).

### Significant Differences in Gene Mutation, CNV, and Clinical Characteristics Between the High- and Low-FE.score Groups

**FIGURE 7:**
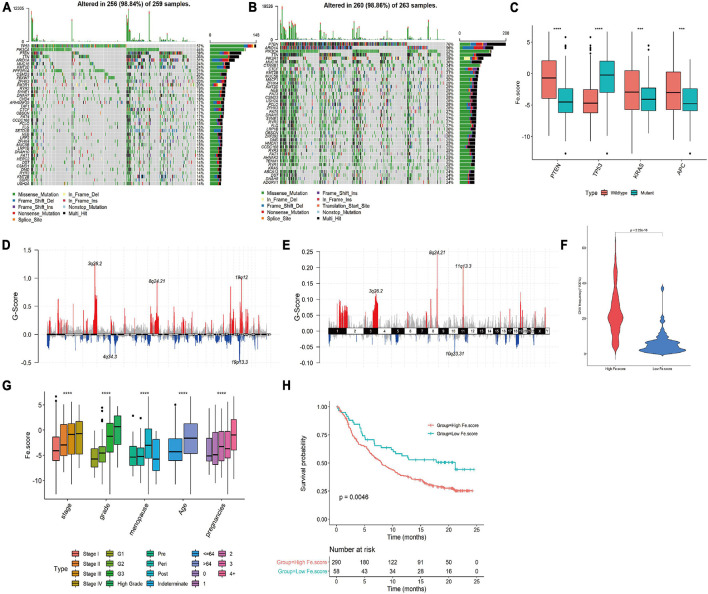
Correlation between ferroptosis score and clinical characteristics. **(A)** Somatic mutation spectrum of the high-Fe.score group. **(B)** Somatic mutation spectrum of the low-Fe.score group. **(C)** Correlation between PTEN, TP53, KRAS, APC mutation, and Fe.score. **(D)** CNV peak map of the high-Fe.score group. **(E)** CNV peak map of the low-Fe.score group. The difference in Fe.score for each clinical indicator (stage, grade, menopausal status, age, and fertility status) in endometrial cancer samples. **(H)** Kaplan–Meier survival curve of Fe.score. **(F)** Violin chart of CNV mutation frequency in High Fe.score group and Low Fe.score group. **(G)** The difference of Fe.score in clinical indicators in EC samples. ***p < 0.001 and ****p < 0.0001.

The somatic mutation spectrum of the High Fe.score group is shown in Figure 7A, and the somatic mutation spectrum of the Low Fe.score group is shown in Figure 7B. The mutated genes and the types of mutations in the two groups were significantly different. We found that PTEN mutation, TP53 wild-type, KRAS mutation, and APC mutation patients had lower Fe.scores (Figure 7C). Figures 7D,E show the somatic mutation spectrum of the high- and low-Fe.score groups, and the G-score of the low-Fe.score group is significantly lower than that of the high-Fe.score group; the CNV frequency of the low-Fe.score group is also significantly lower than that of the high-Fe.score group (Figure 7F).

For clinical indicators, we found that patients with stage I, G1, premenopausal, younger age, and fewer children had lower Fe.scores (Figure 7G). The Kaplan–Meier survival curve of the Fe.score shows that there is a very significant difference in survival between the high-Fe.score and low-Fe.score groups, and the low-Fe.score group has a significant survival advantage (see Figure 7H).

### Fe.score for Predicting the Effect of Immunotherapy and Prognosis

**FIGURE 8:**
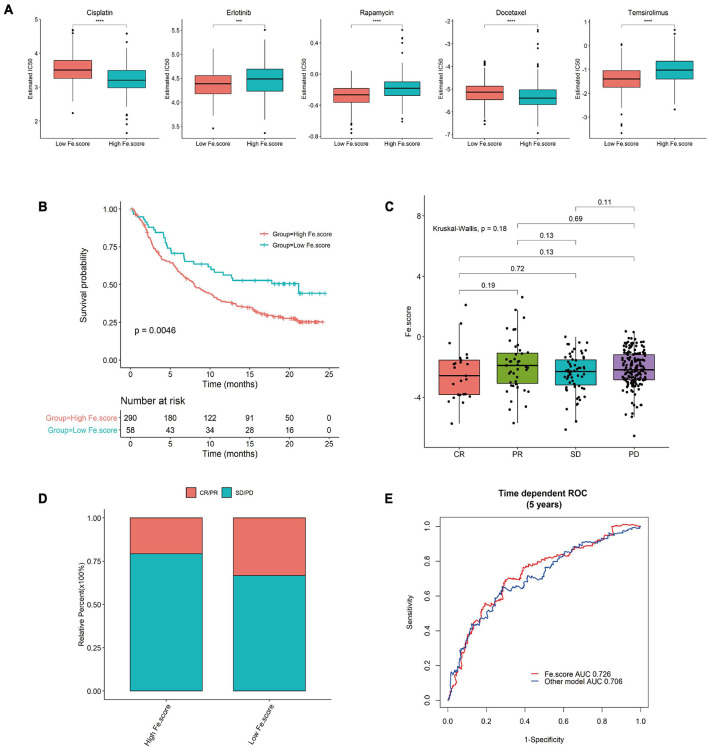
Immunotherapy results in the high- and low-Fe.score groups. **(A)** GDSC predicts the IC50 difference of five drugs between Fe.score groups. **(B)** Kaplan–Meier survival curve between Fe.score groups in the bladder cancer dataset (IMvigor210). **(C)** The proportion of CR/PR and SD/PD between Fe.score groups. **(D)** The differences in Fe.score among CR, PR, SD, and PD. **(E)** Fe.score 5-year receiver operating characteristic curve compared with other prognostic models of endometrial cancer.

We used GDSC to predict the difference in IC50 of the drugs cisplatin, erlotinib, rapamycin, docetaxel, and temsirolimus between the Fe.score groups. Through the nonparametric Wilcoxon rank sum test, the differences reached a very significant level (Figure 8A). We calculated and grouped the Fe.score of the bladder cancer dataset (IMvigor210) and found that the low-Fe.score group had a very significant survival advantage (Figure 8B). The Fe.score difference between the CR, PR, SD, and PD groups was not significant (Figure 8C), and CR/PR patients accounted for a relatively high proportion. Chi-square tests were performed on CR/PR and SD/PD between the high-Fe.score and low-Fe.score groups, and the difference was not significant (*p*-value = 0.075) (Figure 8D). Figure 8E shows that the Fe.score is better than the previous prognostic model of endometrial carcinoma (35), and the AUC value is higher.

## Discussion

In this article, we first explored the expression and mutation of ferroptosis-related genes in normal and EC tissues. According to the different expression levels of related genes, 544 EC samples were divided into four clusters, and most of the infiltrating immune cells were significantly different among the four groups. The tumor stages and grades of Fe.cluster A and Fe.cluster B were lower, and the enrichment scores of angiogenesis, CD8 T effector, EMT2, and panfibroblast TGFb were significantly different among Fe.clusters. A gene expression difference analysis between Fe.cluster groups was performed, and 438 overlap difference genes were obtained by taking the intersection. According to the difference genes, the samples were again divided into three Fe.gene.clusters. Fe.gene.cluster A and Fe.gene.cluster B have survival advantages. Finally, 13 characteristic genes were selected as ferroptosis gene signatures, and the Fe.score was obtained by calculation. A low Fe.score has a significant survival advantage, and GDSC predicts that the IC50 of multiple chemotherapeutic drugs is also significantly different between the two groups. The low-Fe.score group in the bladder cancer data set also had a very significant survival advantage, and CR/PR patients accounted for a relatively high proportion. Compared with previous prognostic models of EC, our prognostic model based on ferroptosis genes is more accurate and simpler (Deng et al., 2020).

Ferroptosis is regulated cell death that distinguishes apoptosis and oxidative damage. The process is controlled by a variety of molecular signaling pathways through epigenetic, transcription, and posttranslational mechanisms (Chen et al., 2020). Iron has a unique role and function in the female reproductive system, and iron disorders are found in many gynecological diseases (Ng et al., 2020). According to reports, iron-mediated cell death (ferroptosis) is closely related to endometriosis, repeated implantation failure, endometrial hyperplasia, and many other endometrial diseases, which can be used as treatment target (Bielfeld et al., 2019; Ng et al., 2020; Zhang et al., 2021). However, the role of ferroptosis in EC remains unclear. In our study, we divided the EC samples into four clusters based on the differences in expression levels of 60 ferroptosis-related genes. These 60 related genes were all verified to be closely related to ferroptosis, such as prostaglandin-endoperoxide synthase 2 (PTGS2/COX2), which is the most upregulated gene among 83 oxidative stress-related genes in BJeLR cells after treatment with erastin or RSL3 and is used as a pharmacodynamic marker for mast cell tissue in mice exposed to erastin or RSL3 (Yang et al., 2014); ChaC glutathione-specific gamma-glutamylcyclotransferase 1 (CHAC1/BOTCH) is the most upregulated gene after treatment with systemic xc^–^ inhibitors *in vitro* and provides a selective pharmacodynamic marker for ferroptosis induced by system xc^–^ inhibitors (Dixon et al., 2014). After we grouped the samples with 60 ferroptosis-related genes, we were able to observe significant differences in tumor grade, stage, functions such as angiogenesis, and EMT between the groups, indicating that the ferroptosis process plays an important role in the occurrence and development of EC.

The relationship between ferroptosis and the tumor immune microenvironment remains elusive. To carefully study the tumor immune microenvironment of EC, we performed ssGSEA to evaluate the abundance of immune cells in different Fe.clusters and the relationship between immune cells and prognosis. We found that, in different Fe.clusters, there were significant differences in the degree of infiltration of 21 types of immune cells. This showed that there was heterogeneity in the immune response between tumors, and ferroptosis is likely to play an important role in it (Dou et al., 2020). Ferroptosis releases damage-related molecular patterns that can be sensed by immune cells to amplify the inflammatory response. With the advent of immunotherapy, people are increasingly aware of the impact of the immune microenvironment on cancer behavior and clinical outcomes (López-Janeiro et al., 2021). Furthermore, we found that activated CD8 T cells, eosinophils, CD56dim natural killer cells, and activated B cells are good prognostic factors for EC, which is basically consistent with the research results of the previous research (López-Janeiro et al., 2021). Activated CD8 T cells and eosinophils have been widely verified to have significant antitumor effects (Sakkal et al., 2016; Loo Yau et al., 2021; Pauken et al., 2021). On the other hand, some reports have demonstrated reduced CD8 expression in cytotoxic tumor-infiltrating T cells, which could limit antigen presentation and adaptative immune response in EC (Pascual-García et al., 2016). Our results showed the importance of immune cells in EC, which may guide future immunotherapy strategies in these specific tumor subtypes.

We further analyzed the differentially expressed genes in Fe.clusters and divided the samples into different Fe.gene.clusters based on these genes. Univariate Cox regression analysis obtained 204 prognosis-related genes, and through the random forest model, we finally screened 13 feature genes (TUBB4A, TMPRSS2, STX18, LINC01224, SLC25A35, CD7, COL23A1, ZG16B, KCNK6, NWD1, C11orf63, GZMM, and NMU) as ferroptosis gene signatures. The current research shows that most of these 13 genes are closely related to the occurrence and development of tumors, like the TMPRSS2 gene, which is abnormally expressed in approximately 50% of cases of prostate cancer and is a key driver of prostate oncogenesis (Hong et al., 2020); COL23A1 plays an oncogenic role in clear cell renal cell carcinoma (Xu et al., 2017), and LINC01224 is also closely related to hepatocellular carcinoma and ovarian cancer (Gong et al., 2020; Xing et al., 2020). However, at present, the roles of these genes in EC are still unclear; whether these genes are involved in the pathogenesis of EC through ferroptosis pathways and affect prognosis may be worth studying in the future. Finally, through PCA calculation, we obtained the Fe.score, and we verified that it is a simple and effective prognostic indicator, which is better than the previous prognostic model in EC (Deng et al., 2020). The FIGO staging system and the histological typing are the most commonly adopted classification for the treatment and prognosis for EC patients, but there are still limitations (Yang et al., 2016). Remarkably, a significant association has been observed between our model and many clinical and molecular features. This model can be further combined with FIGO staging and/or other histological classifications to have more powerful prognostic prediction capabilities and may also be an alternative or complementary method for the molecular classification of EC. It may also contribute to reasonable treatment and avoid under- or overtreatment.

There are still some flaws in this study. When Fe.cluster was divided into four categories, the overlapping difference genes have only single digits, so the Fe.cluster D group has to be removed before the difference analysis is performed. Fe.cluster D had only a few samples, the expression of some genes (PGD, G6PD, AKR1C1, AKR1C2, AKR1C3, and NQO1) was particularly high, and some clinical indicators, such as BMI, were very different from the other three clusters. However, whether Fe.cluster D truly represents a special subtype with the same clinical or molecular characteristics still needs to be verified by more samples. When we perform ferroptosis score calculation, we did not consider the negative eigen values. We hope that we can re-establish the model and reanalyze the negative eigen values in the future. In addition, since there are no immunotherapy results for EC, we used bladder cancer immunotherapy data (large sample size) to evaluate whether the Fe.score can predict the effect of immunotherapy. Although our results were validated in the bladder cancer data set and the previous literature has also verified that using other cancer data sets is feasible (Wang et al., 2020; Zhang X. et al., 2020), we hope that there will be EC data sets in the future to verify our model.

## Conclusion

Fe.score can not only reflect the immunological and carcinogenic status but also predict the prognosis of patients. In addition, Fe.score provides additional prognostic value for existing FIGO and molecular subtypes. In conclusion, our research has explored the relationship between EC and ferroptosis in detail, provides comprehensive insights for ferroptosis-mediated EC mechanism research, and emphasizes the clinical application potential of Fe.score-based immunotherapy strategies.

## Data Availability Statement

The original contributions presented in the study are included in the article/Supplementary Material, further inquiries can be directed to the corresponding author/s.

## Author Contributions

HW and MX contributed to the conceptualization. YW participated in data curation. HW and YW participated in formal analysis. MH contributed to the methodology. HW took charge of the software. HW, YW, MH, SC, and YY participated in writing – original draft. MX participated in writing – review and editing. All authors contributed to the article and approved the submitted version.

## Conflict of Interest

The authors declare that the research was conducted in the absence of any commercial or financial relationships that could be construed as a potential conflict of interest.

## Publisher’s Note

All claims expressed in this article are solely those of the authors and do not necessarily represent those of their affiliated organizations, or those of the publisher, the editors and the reviewers. Any product that may be evaluated in this article, or claim that may be made by its manufacturer, is not guaranteed or endorsed by the publisher.
